# Taste and Smell Impairment in COVID-19: An AAO-HNS Anosmia Reporting Tool-Based Comparative Study

**DOI:** 10.1177/0194599820931820

**Published:** 2020-06-09

**Authors:** İbrahim Sayin, Kadriye Kart Yaşar, Zahide Mine Yazici

**Affiliations:** 1 Department of Otolaryngology Head and Neck Surgery, Bakırköy Dr. Sadi Konuk Teaching and Research Hospital, Istanbul, Turkey; 2 Department of Infectious Diseases, Bakırköy Dr. Sadi Konuk Teaching and Research Hospital, Istanbul, Turkey

**Keywords:** COVID-19, coronavirus, chemosensory, anosmia, dysgeusia

## Abstract

**Objective:**

To identify the taste and smell impairment in coronavirus disease 2019 (COVID-19)–positive subjects and compare the findings with COVID-19–negative subjects using the American Academy of Otolaryngology–Head and Neck Surgery (AAO-HNS) Anosmia Reporting Tool.

**Setting:**

Tertiary referral center/COVID-19 pandemic hospital.

**Study Design:**

Comparative study.

**Subjects and Methods:**

After power analysis, 128 subjects were divided into 2 groups according to real-time polymerase chain reaction (RT-PCR) COVID-19 testing results. Subjects were called via telephone, and the AAO-HNS Anosmia Reporting Tool was used to collect responses.

**Results:**

The mean age of the study group was 38.63 ± 10.08 years. At the time of sampling, rhinorrhea was significantly high in the COVID-19–negative group, whereas those complaints described as “other” were significantly high in the COVID-19–positive group. There was a significant difference in the smell/taste impairment rates of the groups (n = 46% [71.9%] for the COVID-19–positive group vs n = 17 [26.6%] for the COVID-19–negative group, *P* = .001). For subjects with a smell impairment, anosmia rates did not differ between the groups. The rates of hyposmia and parosmia were significantly high in the COVID-19–positive group. For the subjects with taste impairment, ageusia rates did not differ between groups. The rate of hypogeusia and dysgeusia was significantly high in the COVID-19–positive group. Logistic regression analysis indicates that smell/taste impairment in COVID-19–positive subjects increases the odds ratio by 6.956 (95% CI, 3.16-15.29) times.

**Conclusion:**

COVID-19–positive subjects are strongly associated with smell/taste impairment.

The world was faced with a novel form of coronavirus, named coronavirus disease 2019 (COVID-19), by the World Health Organization (WHO) in December 2019. According to the Coronavirus Study Group (CSG) of the International Committee, the virus became known as severe acute respiratory syndrome coronavirus 2 (SARS-CoV-2).^1^

Since there is no specific treatment or effective vaccine, the outbreak can only be controlled by strict isolation and hygiene rules. COVID-19 is a form of upper respiratory tract infection (URTI), and the clinical course of every URTI is different, so presenting and alarm symptoms are needed to restrict transmission.^2^

In the early phases of the outbreak, fever, dyspnea, coughing, and travel to endemic counties were used as the main screening parameters. However, during the course of the outbreak, different symptomatology, including headache, sore throat, nasal congestion, rhinorrhea, fatigue, tonsil swelling, and conjunctivitis, began to be published.^3^ Among these, frequent chemosensory involvement, as evaluated by smell and taste dysfunctions, was reported.^4-6^

During the outbreak, many authors reported an increase in the presence of anosmia in COVID-19 subjects. After initial reports, a few studies indicated that anosmia presenting in COVID-19 subjects is more frequent than expected for a routine upper respiratory tract infection. Although most of these reports were not comparative, these findings are also in the Centers for Disease Control and Prevention (CDC) list of symptoms, and according to the CDC, “new loss of taste or smell” can occur within 2 to 14 days of exposure.^7^ Chemosensory involvement can also occur as a first sign of the disease. This is important because, given that there is no specific treatment or effective vaccine, the outbreak can only be controlled by strict isolation and hygiene measures.^1,2^ In addition, these subjects are potential ear, nose, and throat (ENT) clinic patients in the future for sustained taste and smell loss. The clinical course, along with other symptoms and their timing, requires specialist monitoring to identify its properties. Clarifying the chemosensory involvement not only provides valuable data for clinical evaluation but also gives important clinical information of the virus characteristics, which may be used by researchers from different disciplines.

The American Academy of Otolaryngology–Head and Neck Surgery (AAO-HNS) has been continuously screening the outbreak and providing otolaryngology–head and neck surgery specialists with information about various aspects of the outbreak. As a result of cumulative anecdotal evidence of anosmia/dysgeusia around the world during the outbreak, the COVID-19 Anosmia Reporting Tool was developed in March 2020 by the AAO-HNS.^8^ This tool was developed by 2 committees of the AAO-HNS: the Infectious Disease and Patient Safety Quality Improvement committees. The tool is completed online either by the medical provider or the patient and consists of 17 questions relating to demographic factors, COVID status, risk factors, symptoms, and onset of anosmia/dysgeusia and so on and may be found at https://www.entnet.org/content/reporting-tool-patients-anosmia-related-covid-19. This anosmia reporting tool collects data to establish the importance of smell and taste impairment in the clinical course of COVID-19. For this purpose, we used the tool and conducted a comparative study with COVID-19–negative subjects.

## Methods

This study was conducted at Bakırköy Dr.Sadi Konuk Teaching and Research Hospital, with ethical approval obtained from its local ethics committee. Subjects with upper respiratory tract infection symptoms who had been assessed as COVID-19 positive were enrolled. All subjects underwent real-time polymerase chain reaction (RT-PCR) testing.

The exclusion criteria included subjects younger than age 18 years and those without upper respiratory tract infection symptoms. These subjects had undergone routine COVID-19 testing as relatives of COVID-19 subjects and health care workers. In addition, the exclusion criteria included subjects with pending results, those who had tested negative for COVID-19 but were regarded as COVID-19 positive based on their clinical presentations (presumed positive), and subjects who had to remain in an intensive care unit.

Subjects were divided into 2 groups: COVID-19 positive (group A) and COVID-19 negative (group B) according to RT-PCR results. Two questionnaires were prepared, based on the AAO-HNS Anosmia Reporting Tool, for groups A and B, respectively.

According to power analysis, 64 subjects were required for each group. All questionnaires were uploaded to Google Forms, and subjects were admitted to clinics and the hospital began screening. All subjects were contacted by telephone and asked to confirm they fulfilled the inclusion criteria. Where the subject fulfilled the inclusion criteria and informed consent was then obtained, the questionnaire was completed with the assistance of physicians and recorded to Google Forms.

### Questionnaires

#### Questionnaire of COVID-19–Positive Subjects (Supplemental Questionnaire 1)

The AAO-HNS Anosmia Reporting Tool was used to evaluate subjects.^8^ The questionnaire included 17 questions, with 1 question asking about both anosmia and dysgeusia. To obtain more detailed data, we expanded this question and separated anosmia and dysgeusia questions from each other. Subjects were asked to identify the type of smell and taste impairments separately, with a smell impairment subgrouped as anosmia, hyposmia, and parosmia and a taste impairment subgrouped as ageusia, hypogeusia, and dysgeusia. If hyposmia or hypogeusia was reported, then subjects were asked to rate the relative decrease in taste and smell on a 10-point visual analog scale (VAS).

#### Questionnaire for COVID-19–Negative Subjects (Supplemental Questionnaire 2)

The AAO-HNS Anosmia Reporting Tool was issued to subjects with COVID-19. This study also contains a subject group with COVID-19–negative test results, and to maintain consistency and comparability of findings, questions relating to the presence of COVID-19 were removed and questionnaire 2 was created.

### Statistical Analysis

NCSS (Number Cruncher Statistical System) 2007 software was used for the statistical analysis of the results, with descriptive statistical methods (mean, standard deviation, median, frequency, ratio, minimum and maximum) used when evaluating the study data. The suitability of quantitative data for normal distribution was tested using the Shapiro-Wilk test and graphical evaluations. A Student *t* test was used to compare 2 groups of normal distribution variables, and a Pearson χ^2^ test and Fisher exact test were used for comparison of qualitative data with normal distribution. Logistic regression analysis was used in the multivariate analysis. Significance was evaluated at *P* < .05.

### Power Analysis

To determine the sample size, power analysis was performed using G*Power (v3.1.7). According to Cohen effect size coefficients, assuming the evaluation being made between 2 independent groups will have a medium effect size (*d* = 0.5), the results showed that a total sample of 128 participants with 2 equally sized groups of 64 would be required to achieve a power of .80.

## Results

**Figure 1.**
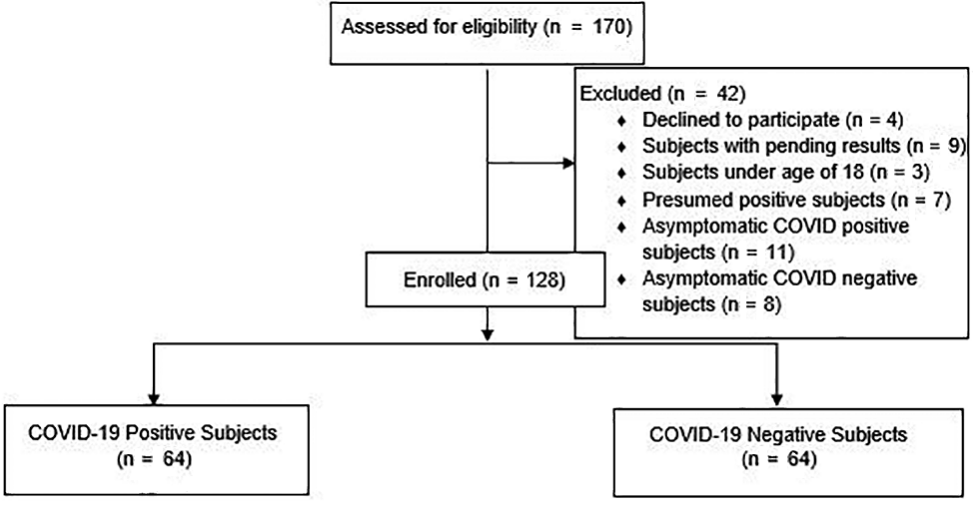
Flowchart of the study.

In total, 170 subjects were screened for the study. Forty-two of 170 subjects did not meet the inclusion criteria. Finally, 128 subjects were enrolled (**Figure 1** shows a flowchart of the study). Both COVID-19–positive and COVID-19–negative groups consisted of 64 subjects each, with 48 (37.5%) out of the total 128 subjects being male and the remaining 80 subjects being female. The mean age of the study group was 38.63 ± 10.08 (range, 21-77).

The groups did not differ in terms of age, sex, risk factors for COVID-19 infection, and comorbidities (*P* > .05 for all comparisons) (**Table 1**). At the time of sampling, only rhinorrhea and those complaints described as “other” were significantly different between the 2 groups. Rhinorrhea was significantly high in the COVID-19–negative group (n = 21 [32.8%] for COVID-19–negative group vs n = 11 [17.2%] for COVID-19–positive group, *P* = .041), whereas “other” complaints were significantly high in the COVID-19–positive groups (n = 12 [18.8%] for COVID-19–positive group vs n = 4 [6.3%] for COVID-19–negative group, *P* = .033).

**Table 1.** Age, Sex, Risk Factors for COVID-19 Infection, Comorbidities, and Complaints of Subjects Who Underwent Smell/Taste Evaluation via AAO-HNS Anosmia Reporting Tool.

Characteristic	COVID-19 Positive (n = 64)	COVID-19 Negative (n = 64)	*P* Value
Age, y			
Minimum-maximum (median)	21-77 (39)	22-62 (40.5)	.341^a^
Mean ± SD	37.78 ± 11.34	39.48 ± 8.64	
Sex, No. (%)			
Male	25 (39.1)	23 (35.9)	.715^b^
Female	39 (60.9)	41 (64.1)	
Risk factors for COVID-19 infection, No. (%)^c^			
Health care worker	50 (78.1)	52 (81.3)	.660^b^
Close contact with a confirmed case	34 (53.1)	41 (64.1)	.209^b^
Congregant living	2 (3.1)	3 (4.7)	1.000^d^
Travel to known areas with widespread community transmission	1 (1.6)	1 (1.6)	1.000^d^
Risk factors/comorbidities, No. (%)^c^			
Smoking	14 (21.9)	21 (32.8)	.165^b^
Head trauma	9 (14.1)	14 (21.9)	.250^b^
Sinusitis/allergy	9 (14.1)	10 (15.6)	.804^b^
Chronic respiratory disease/asthma	4 (6.3)	3 (4.7)	1.000^d^
Cardiac disease	2 (3.1)	1 (1.6)	1.000^d^
Other	5 (7.8)	11 (17.2)	.109^b^
Complaints when the sample is received from the patients, No. (%)^c^			
Fever	18 (28.1)	24 (37.5)	.259^b^
Chills	19 (29.7)	11 (17.2)	.095^b^
Malaise	41 (64.1)	35 (54.7)	.280^b^
Cough	28 (43.8)	38 (59.4)	.077^b^
Headache	20 (31.3)	29 (45.3)	.102^b^
Nasal congestion	18 (28.1)	19 (29.7)	.845^b^
Rhinorrhea	11 (17.2)	21 (32.8)	.041^b,e^
Gastrointestinal distress	11 (17.2)	15 (23.4)	.380^b^
Pneumonia	15 (23.4)	12 (18.8)	.516^b^
Other	12 (18.8)	4 (6.3)	.033^b,e^

Abbreviations: COVID-19, coronavirus disease 2019; VAS, visual analog scale.

a Student *t* test.

b Pearson χ^2^ test.

c More than 1 answer exists.

d Fisher exact test.

e *P* < .05.

For COVID-19–positive subjects, in 28 (43.8%) of the 64 subjects, the source of infection was not known. In 46 (71.9%) of the 64 subjects, the COVID-19 infection status was active, and in 34 (53.1%) out of 64 subjects, the smell/taste impairment was observed before diagnosis. All subjects received treatment for COVID-19 (n = 64 [100%]). Fifty (78.1%) of 64 subjects were treated on an outpatient basis, and the remaining 14 (21.9%) subjects were treated as inpatients (**Table 2**).

**Table 2.** COVID-19–Positive Subjects’ Data According to Source, Current Status of the Infection, and Onset of Smell/Taste Impairment.

COVID-19–Positive Subjects	No. (%)
Is the source of the COVID-19 infection identifiable?	
Yes	28 (43.8)
No	36 (56.3)
What is the patient’s current COVID-19 infection status?	
Active	46 (71.9)
Recovered	18 (28.1)
Did the patient receive treatment?	
Yes	64 (100)
No	0 (0)
When was the anosmia (loss of sense of smell) or dysgeusia (alteration of sense of taste) first noticed by the patient?	
Before diagnosis	34 (53.1)
After diagnosis	12 (18.8)
No impairment in smell/taste	18 (28.1)
What was the condition of the COVID-19 infection at the time the smell/taste impairment was observed?	
Inpatient/hospitalized	14 (21.9)
Outpatient	50 (78.1)

Abbreviation: COVID-19, coronavirus disease 2019.

There was a significant difference between the smell/taste impairment rates between the groups (n = 46 [71.9%] for the COVID-19–positive group vs n = 17 [26.6%] for the COVID-19–negative group, *P* = .001) (**Table 3**).

**Table 3.** Comparison of Smell/Taste Impairment Between Groups.

Characteristic	COVID-19 Positive (n = 64)	COVID-19 Negative (n = 64)	*P* Value
Did the patient have smell/taste impairment? No. (%)			
Absent	18 (28.1)	47 (73.4)	.001^a,b^
Present	46 (71.9)	17 (26.6)	
Definition of smell impairment, No. (%)^c^			
Anosmia	8 (12.5)	3 (4.7)	.115^a^
Hyposmia	33 (51.6)	10 (15.6)	.001^a,b^
Parosmia	11 (17.2)	2 (3.1)	.008^a,b^
Normal	21 (32.8)	51 (79.7)	.001^a,b^
VAS of subjects with hyposmia			
Minimum-maximum (median)	2-9 (5)	2-9 (7.5)	.049^d,e^
Mean ± SD	5.48 ± 2.18	7.00 ± 2.05	
Definition of taste impairment, No. (%)^c^			
Ageusia	8 (12.5)	3 (4.7)	.115^a^
Hypogeusia	36 (56.3)	10 (15.6)	.001^a,b^
Dysgeusia	16 (25.0)	4 (6.3)	.003^a,b^
Normal	18 (28.1)	49 (76.6)	.001^a,b^
VAS of subjects with hypogeusia			
Minimum-maximum (median)	2-9 (5)	3-9 (7.5)	.145^e^
Mean ± SD	5.61 ± 2.09	6.70 ± 2.26	
Any other symptoms before the development of smell/taste impairment? No. (%)			
Yes	32 (69.6)	15 (88.2)	.195^f^
No	14 (30.4)	2 (11.8)	
What symptoms did the patient have at the time of smell/taste impairment? No. (%)^c^			
Fever	11 (23.9)	7 (41.2)	.216^f^
Chills	14 (30.4)	2 (11.8)	.195^f^
Malaise	24 (52.2)	9 (52.9)	.957^a^
Cough	18 (39.1)	10 (58.8)	.163^a^
Headache	18 (39.1)	9 (52.9)	.325^a^
Nasal congestion	11 (23.9)	5 (29.4)	.747^f^
Rhinorrhea	6 (13.0)	5 (29.4)	.149^f^
Gastrointestinal	6 (13.0)	4 (23.5)	.438^f^
Pneumonia	9 (19.6)	5 (29.4)	.498^f^
Other	4 (8.7)	1 (5.9)	1.000^f^
Did the patient’s condition worsen or improve after the smell/taste impairment was observed? No. (%)			
Worsen	19 (41.3)	4 (23.5)	.193^a^
Improved	27 (58.7)	13 (76.5)	
Did the smell/taste impairment resolve? No. (%)			
Yes	21 (45.7)	11 (64.7)	.179^a^
No	25 (54.3)	6 (353)	

Abbreviations: COVID-19, coronavirus disease 2019; VAS, visual analog scale.

a Pearson χ^2^ test.

b *P* < .01.

c More than one answer exists.

d *P* < .05.

e Mann-Whitney *U* test.

f Fisher exact test.

For subjects with smell impairment, anosmia rates did not differ between the groups (n = 8 [12.5%] for the COVID-19–positive group vs n = 3 [4.7%] for the COVID-19–negative group, *P* = .115). The rates of hyposmia (n = 33 [51.6%] for the COVID-19–positive group vs n = 10 [15.6%] for the COVID-19–negative group, *P* = .001) and parosmia (n = 11 [17.2%] for the COVID-19–positive group vs n = 2 [3.1%] for the COVID-19–negative group, *P* = .008) were significantly high in the COVID-19–positive group. For subjects with hyposmia, VAS scales were significantly lower within the COVID-19–positive group (5.48 ± 2.18 for the COVID-19–positive group vs 7.00 ± 2.05 for the COVID-19–negative group, *P* = .049).

For the subjects with a taste impairment, ageusia rates did not differ between the groups (n = 8 [12.5%] for the COVID-19–positive group vs n = 3 [4.7%] for the COVID-19–negative group, *P* = .115). The rates of hypogeusia (n = 36 [56.3%] for the COVID-19–positive group vs n = 10 [15.6%] for COVID-19–negative group, *P* = .001) and dysgeusia (n = 16 [25%] for the COVID-19–positive group vs n = 4 [6.3%] for the COVID-19–negative group, *P* = .003) were significantly high in the COVID-19–positive group. For subjects with hypogeusia, VAS scales did not differ between the groups (5.61 ± 2.09 for the COVID-19–positive group vs 6.70 ± 2.26 for the COVID-19–negative group, *P* = .145).

In the COVID-19–positive group, 3 (4.7%) subjects reported isolated taste impairment. In the COVID-19–negative group, 4 (6.2%) subjects reported isolated taste impairment and 2 (3.1%) subjects reported isolated smell impairment.

Symptoms at the time of smell/taste impairment, symptoms before the development of smell/taste impairment, patient’s condition observed after the smell/taste impairment, and smell/taste impairment resolution rates were not significantly different between groups (*P* > .05 for all comparisons).

When we evaluate the factors affecting the COVID-19–positive subjects with backward logistic regression analysis, the model was found to be significant and explanatory. The coefficient of the model (72.7%) was good. It was identified that the effect of the taste and smell impairment on COVID-19–positive subjects increases the odds ratio by 6.956 (95% CI, 3.16-15.29) times. Rhinorrhea is also close to the limit of significance but not significant. No other significant factor was determined in multivariate analysis.

## Discussion

In a power-analyzed comparative setting, we included 128 subjects for the study. These were in 2 groups (COVID-19 positive and COVID-19 negative) consisting of 64 subjects each. The test results were based on RT-PCR testing. Both groups underwent assessment via the anosmia reporting tool developed by AAO-HNS in March 2020. When compared with the COVID-19–negative subjects, multivariate analysis indicates that subjects testing positive for COVID-19 experienced taste and smell impairment approximately 7 times higher than those testing negative for COVID-19. Taste and smell impairment occurred mainly in the forms of hyposmia/hypogeusia.

COVID-19–related smell impairments were initially outlined in anecdotal reports and the experiences of physicians around the world.^6^ Immediately after these reports, the AAO-HNS released an anosmia reporting tool to collect data and to clarify this observational finding.^8^ The tool was modified a few times but serves as a basis for systematic evaluation of the situation. Following the publication of the initial results from the reporting tool, of the first 237, in 26.6% of the subjects, anosmia was the initial symptom of COVID-19.^9^ In 73% of the subjects, anosmia was reported occurring before diagnosis and 85% of the subjects who reported anosmia improved in the first 10 days, indicating that anosmia can be a presenting symptom with good spontaneous recovery rate.

We conducted our study according to this tool, as it allows for a precise evaluation of subjects. To identify the subjects’ smell and taste impairment in detail, we changed the “anosmia/dysgeusia” term to “smell/taste impairment” for all related questions. In addition, we expanded the question “Did the patient have smell/taste impairment?” and asked subjects to identify their impairment as anosmia/hyposmia/parosmia for a smell impairment and as ageusia/hypogeusia/dysgeusia for a taste impairment. Where the subject reported hyposmia or hypogeusia, they were then asked to indicate their loss on a 10-point VAS scale. This expansion allowed us to evaluate the type of smell/taste impairment in much greater detail. For subjects testing negative for COVID-19, a second questionnaire was developed; given that the anosmia reporting tool contained some questions for subjects with COVID-19, these questions were not applicable for the subjects testing negative for COVID-19. This second questionnaire is similar to the anosmia reporting tool; however, questions relating to COVID-19 presence have been removed. The reason we used the anosmia reporting tool as a control is to provide comparability and internal consistency within the study.

Our results indicate that the COVID-19–positive subjects experienced significantly higher smell/taste impairment than COVID-19–negative subjects. However, most of these impairments were not in the anosmia/ageusia form but rather in the hyposmia/hypogeusia and parosmia/dysgeusia form. Although not significant between groups, at the time of study, 45.7% of the subjects in the COVID-19–positive group and 64.7% of the subjects in the COVID-19–negative group, taste and smell impairment return to normal. Although not significant in multivariate analysis, the presence of rhinorrhea was significantly high among the COVID-19–negative subjects, and those complaints described as “other” were significantly high among COVID-19–positive subjects. This may indicate a need for more detailed symptomology questions within the anosmia reporting tool.

Recorded levels of smell/taste impairment vary from location to location, with most studies reporting single-arm data on COVID-19–positive subjects. Vaira et al^5^ have reported the incidence of chemosensory dysfunction as 19.4% among the first 320 subjects. However, they noted that this incidence may be low as most subjects were not asked about taste and smell impairment. In a multicenter study within Europe, of 417 respondents, 85.6% and 88.0% of subjects reported smell and taste impairment.^10^ Anosmia was recorded as the first symptom among 11.8% of these subjects. Females were generally more affected, and the early recovery rate was 44%.

Comparative studies may be helpful to differentiate certain disease characteristics. Comparing COVID-19–positive subjects with negative ones may provide clearer comparisons. Yan et al^4^ compared 59 COVID-19–positive subjects with 203 COVID-19–negative subjects. Olfactory impairment (68% for COVID-19–positive subjects vs 16% of COVID-19–negative subjects) and gustatory involvement (71% for COVID positive subjects vs 17% of COVID-19–negative subjects) were both significantly high in COVID-19–positive subjects. Their results indicated that, among these complaints, smell and taste impairment were strongly associated with COVID-19 positivity, and presence of sore throat was independently associated with COVID-19 negativity. COVID-19–positive subjects were 10 times more likely to experience a taste and smell impairment; however, COVID-19–negative subjects were 4 to 5 times more likely to report a sore throat. At the time of the survey, around 29 out of 40 (72.5%) subjects who experienced smell loss improved in 1 month. Yan et al^4^ reported approximately a 2 times higher smell/taste impairment than did Giacomelli et al,^6^ who undertook a study with hospitalized subjects. The authors evaluated 59 hospitalized subjects and reported that 20 out of 59 subjects (33.9%) experienced at least 1 taste or olfactory disorder, and 11 out of 59 (18.6%) experienced both disorders. Yan et al^4^ proposed that 2 forms of the disease may exist, nasal centric and pulmonary centric, according to a comparison of these findings. Hopkins et al^11^ reported that 1 of 6 subjects experienced anosmia as an isolated symptom.

Upper respiratory infections are known etiologically as olfactory loss.^12^ The prevalence following an URTI was reported to be as high as 20% to 40% by some centers.^2^ Current data indicate that SARS-CoV-2 significantly impairs smell and taste functions. Although the exact mechanisms are unknown, a number of hypotheses exist. Coronavirus is known as a neuroinvasive and neurotrophic virus. Sensory olfactory epithelium and respiratory epithelium are the main parts of nasal epithelium. Key genes responsible for SARS-CoV-2 entry are *ACE2* and *TMPRSS2*. Among all epithelial cell lines, nasal epithelial cells have the highest *ACE2*.^13^ This finding may explain why nasal epithelial cells are the viral targets or reservoirs for SARS-CoV-2. However, olfactory sensory neurons/olfactory bulb neurons did not express *ACE2* and *TMPRSS2*. Brann et al^14^ reported that only nonneural cell types (stem cells, *TMPRSS2* support cells, and perivascular cells) express *ACE2*. Instead of direct involvement with olfactory sensory neurons, olfactory involvement relating to SARS-CoV-2 may be due to nonneural cell impairment. The involvement of nonneural structures may result in an inflammatory response and deteriorated signaling of olfactory sensory neurons. In addition, diffuse architectural damage to the olfactory epithelium is also possible; however, an unreported viral receptor mechanism or a central mechanism cannot be ruled out with current data. The olfactory epithelium is in continuation with the brain and serves as a route for brain involvement.^12^

There is no suggested treatment for COVID-19–related olfactory loss, although nasal steroid use may be offered. The European Academy of Allergy and Clinical Immunology (ARIA-EAACI) statement suggested continuation of intranasal corticosteroids in subjects with allergic rhinitis, as cessation of their use was not advised. However, these data need further clarification.^15^

Although we set up our study with a 0.50 effect size, this report presents the experiences of a single institution. In addition, we need to note that the study was made within a limited time, and some of the subjects in the COVID-19–negative group may have had false-negative testing results. At the beginning of the outbreak, subjects older than 65 years, within the country this study was located in, were forbidden to leave their homes and had to remain in isolation. This is why the subjects were generally not within an older age bracket. This lower number of older patients will limit the outcome of the study. Our study will expand to the analysis of subgroups and may be regarded as a pilot for future studies. In addition, assessment of subjects using objective testing methods is required; however, due to the high transmission possibility of the disease, this is not possible at this time.

## Conclusion

In the past few months, all subspecialties have been working to identify the various aspects of COVID-19 in patients. Among other symptoms, taste and smell impairment have emerged as potential screening symptoms, as research findings strongly suggest that subjects diagnosed as COVID-19 positive can present with smell and taste impairment.

## Implications of Practice

Smell/taste impairment is 7 times higher in subjects testing positive for COVID-19, and as a result, it may be considered useful as a screening tool. The AAO-HNS anosmia reporting tool is a brief, concise, and easy to use tool that can be generalized to collect data around the world.

## Supplemental Material

Supplemental_Questioannare_1 – Supplemental material for Taste and Smell Impairment in COVID-19: An AAO-HNS Anosmia Reporting Tool-Based Comparative Study Click here for additional data file. Supplemental material, Supplemental_Questioannare_1 for Taste and Smell Impairment in COVID-19: An AAO-HNS Anosmia Reporting Tool-Based Comparative Study by İbrahim Sayin, Kadriye Kart Yaşar and Zahide Mine Yazici in Otolaryngology–Head and Neck Surgery

Supplemental_Questioannare_2 – Supplemental material for Taste and Smell Impairment in COVID-19: An AAO-HNS Anosmia Reporting Tool-Based Comparative Study Click here for additional data file. Supplemental material, Supplemental_Questioannare_2 for Taste and Smell Impairment in COVID-19: An AAO-HNS Anosmia Reporting Tool-Based Comparative Study by İbrahim Sayin, Kadriye Kart Yaşar and Zahide Mine Yazici in Otolaryngology–Head and Neck Surgery
