# Supplementary material for: Taste and Smell Impairment in COVID-19: An AAO-HNS Anosmia Reporting Tool-Based Comparative Study
Source: Otolaryngol Head Neck Surg. 2020 Jun 9;163(3):473–9. doi: 10.1177/0194599820931820 (PMC7284454; doi:10.1177/0194599820931820)
Supplement: Supplemental_Questioannare_1 – Supplemental material for Taste and Smell Impairment in COVID-19: An AAO-HNS Anosmia Reporting Tool-Based Comparative Study [file Supplemental_Questioannare_1.docx]

**Q1. Questionnaire used to evaluate COVID-19 positive subjects (Adopted from the American Academy of Otolaryngology-Head and Neck Surgery COVID-19 Anosmia Reporting tool)**

|  | Questions | Please indicate the answer here |
| --- | --- | --- |
| Q1. | Name/Surname |  |
| Q2. | Phone /e-mail address |  |
| Q3. | Age (numbers) |  |
| Q4. | Gender | - Female •Male |
| Q5. | Please list any risk factors for COVID-19 infection present | - None - Healthcare worker - Close contact with a confirmed case - Homeless - Congregant living (dorms, fraternities/sororities, shelters, jail, prison, skilled nursing, assisted living, adult family home) - Travel to known areas with widespread community transmission - Other |
| Q6. | Other risk factors/comorbidities | - None - Smoking - Head trauma - Sinusitis/allergy - Chronic respiratory disease/Asthma - Cardiac disease - Neurologic disease (e.g. Parkinson’s) - Other |
| Q7. | Complaints when the sample is received from the patients | - None - Fever - Chills - Malaise - Cough - Headache - Nasal congestion - Rhinorrhea - Gastrointestinal distress - Pneumonia - Other |
| Q8. | Is the source of the COVID-19 infection identifiable? | - Yes - No |
| Q9. | What is the patient’s current COVID-19 infection status? | - Active - Recovered |
| Q10 | Did the patient receive treatment? | - Yes - No |
| Q11. | Did the patient have smell/taste impairment? | - Yes - No |
| Q12. | Definition of smell impairment  (ıf present) | - Anosmia - Hyposmia - Parosmia |
| Q12a. | Please indicate the degree of hyposmia on 10 scale VAS (indicate number) | 0 1 2 3 4 5 6 7 8 9 10  (10 indicate normal ) |
| Q13. | Definition of taste impairment  (ıf present) | - Ageuisa - Hypogeusia - Dysgeusia |
| Q13a. | Please indicate the degree of hypogeusia on 10 scale VAS (indicate number) | 0 1 2 3 4 5 6 7 8 9 10  (10 indicate normal ) |
| Q14. | What was the condition of the COVID-19 infection at the time the smell/taste impairment was observed? | - Inpatient /hospitalized - Outpatient |
| Q15. | When was the smell/taste impairment first noticed by the patient | - Before diagnosis - After diagnosis |
| Q16. | Did the patient have any other symptoms before the development of smell/taste impairment? | - Yes - No |
| Q17. | What symptoms did the patient have at the tıme of smell/taste impairment? | - None - Fever - Chills - Malaise - Cough - Headache - Nasal congestion - Rhinorrhea - Gastrointestinal distress - Pneumonia - Other |
| Q18. | Did the patient’s condition worsen or improve after the smell/taste impairment was observed? | - Worsen - Improve |
| Q19. | Did the smell/taste impairment resolve? | - Yes - No |
